# Discovery and Characterization of Human Exonic Transcriptional Regulatory Elements

**DOI:** 10.1371/journal.pone.0046098

**Published:** 2012-09-24

**Authors:** Arshad H. Khan, Andy Lin, Desmond J. Smith

**Affiliations:** Department of Molecular and Medical Pharmacology, David Geffen School of Medicine, University of California Los Angeles, Los Angeles, California, United States of America; University of Turin, Italy

## Abstract

We sought exonic transcriptional regulatory elements by shotgun cloning human cDNA fragments into luciferase reporter vectors and measuring the resulting expression levels in liver cells. We uncovered seven regulatory elements within coding regions and three within 3' untranslated regions (UTRs). Two of the putative regulatory elements were enhancers and eight were silencers. The regulatory elements were generally but not consistently evolutionarily conserved and also showed a trend toward decreased population diversity. Furthermore, the exonic regulatory elements were enriched in known transcription factor binding sites (TFBSs) and were associated with several histone modifications and transcriptionally relevant chromatin. Evidence was obtained for bidirectional *cis-*regulation of a coding region element within a tubulin gene, TUBA1B, by the transcription factors PPARA and RORA. We estimate that hundreds of exonic transcriptional regulatory elements exist, an unexpected finding that highlights a surprising multi-functionality of sequences in the human genome.

## Introduction

An important key to deciphering the human genome is to identify the regulatory elements that control gene expression. Indeed, disruption of these elements has been linked to a number of human diseases including cancers [1], preaxial polydactyly [2], Van Buchem disease [3], and facioscapulohumeral muscular dystrophy [4]. Nevertheless, the vast majority of regulatory elements remain unidentified. One major hurdle to annotating transcriptional regulatory elements is that they are ubiquitous and are found in both intergenic [3], [5] and intronic regions [5], [6]. More surprisingly, isolated examples of transcriptional regulatory elements have recently been found in exons, both coding [5], [7], [8] and non-coding [9], [10]. These coding regulatory elements, though critically important given their dual function, are poorly understood and almost completely uncatalogued.

Because regulatory elements can be found anywhere in the genome, large-scale, high-throughput screens are needed to identify them efficiently. Genome-wide searches have met with some success by exploiting several features of regulatory elements, for example their enrichment in transcription factor binding sites [11], [12], [13], [14], and their association with histone modifications [11], [13], [15], [16]. Unfortunately, coding regions have the same properties, complicating the identification of regulatory elements within these regions.

Comparative genomics were among the first approaches used to search for functional elements and identified sequences more conserved across species than would be expected by chance [17]. Although successfully used in intergenic regions, this strategy is not viable for finding regulatory elements within coding regions as both types of sequences are expected to be highly conserved and thus indistinguishable. In fact, early genome-wide attempts to identify regulatory elements intentionally masked coding regions [7]. Recently, it has been shown that regulatory elements within coding regions may be even more conserved than flanking coding regions [8], presumably due to dual selective pressure to retain both regulatory and coding function. Whether or not coding regulatory elements are superconserved as a rule is unknown.

The current view of transcriptional regulatory elements is that they are clusters of transcription factor binding sites (TFBSs), which when bound by complexes of transcription factors (TFs) can recruit or block various critical components of the transcriptional machinery such as RNA polymerase II [18]. By identifying such clusters, genome-wide computational methods have been used to predict the locations of 118,000 regulatory elements [12]. However, TFBS sequences are typically short, 5–15 bp, and degenerate, creating a substantial false positive problem when only computational methods are used. Alternatively, TFBSs can be identified genome-wide experimentally, via ChIP-chip or ChIP-seq [11], [13]. Although less efficient and much more laborious than computational methods, these methods can at least verify TF binding to predicted elements.

Histone modification occurs through recruitment of other regulatory factors and is a means by which gene expression can be controlled with greater finesse than simply relying upon sequence alone. Regulatory elements are often associated with particular chromatin states marked by a number of histone variants, particularly those that are methylated and/or acetylated [15], [16], [18]. Genome-wide maps of histone modification have been used to predict a set of 55,000 enhancers [15]. As transcribed regions are themselves associated with their own histone modifications, how these modifications might change in regions of overlap with regulatory elements is unclear.

Since many searches for transcriptional regulatory elements employ surrogate markers, such as chromatin modifications or evolutionary conservation, we have employed direct measures of transcriptional activity using reporter constructs. Here we report an unbiased search for exonic regulatory elements active in liver cells. We expand on previous work in which we evaluated genomic DNA from the ApoE gene cluster on chromosome 19 for regulatory elements [5]. In that investigation, we shotgun cloned DNA into luciferase reporter vectors to assay regulatory activity. In the present study, we interrogated putative regulatory sequences only from exonic DNA. We assessed the properties of the recovered coding regulatory elements by characterizing their degree of evolutionary conservation, TFBS enrichment, GC-content, and association with histone modifications. Coding regulatory elements were not found to be overwhelmingly defined by one feature, so integrated approaches will be required to identify them on a genome-wide scale.

## Results

### cDNA Library Creation and Luciferase Assays

To maximize transcript coverage we pooled mRNA from three human cell lines, C3A (liver), HEK-293 (kidney) and SVGp12 (astrocytes). We then synthesized cDNA from the RNA. Pooling RNA from a diverse variety of cell types was an attempt to normalize the cDNA library. Because we were only interested in exonic sequences, we restricted our assays to cDNA rather than whole genomic DNA. Pooled cDNA was digested either with Sau3AI or AluI and subcloned into the multiple cloning site upstream of the basal SV40 early promoter of the pGL3-promoter vector. A total of 1,932 clones were created, 1,008 from Sau3AI and 924 from AluI, with an average fragment size of ∼167 bp based on sequencing.

All clone-containing firefly luciferase vectors were co-transfected with *Renilla* luciferase vectors into C3A cells in 96-well plates. Expression of the two luciferase channels was assayed independently, and the regulatory activity of the putative element estimated from the log_10_ ratio of firefly to *Renilla* reporter gene activity. This measure evaluated expression of the tested element relative to transfection efficiency. Transfection efficiency measured using a CMV-GFP construct (pEGFP-N3, Clontech) was uniform at approximately seven percent [5].

### Screening for Regulatory Elements

**Figure 1:**
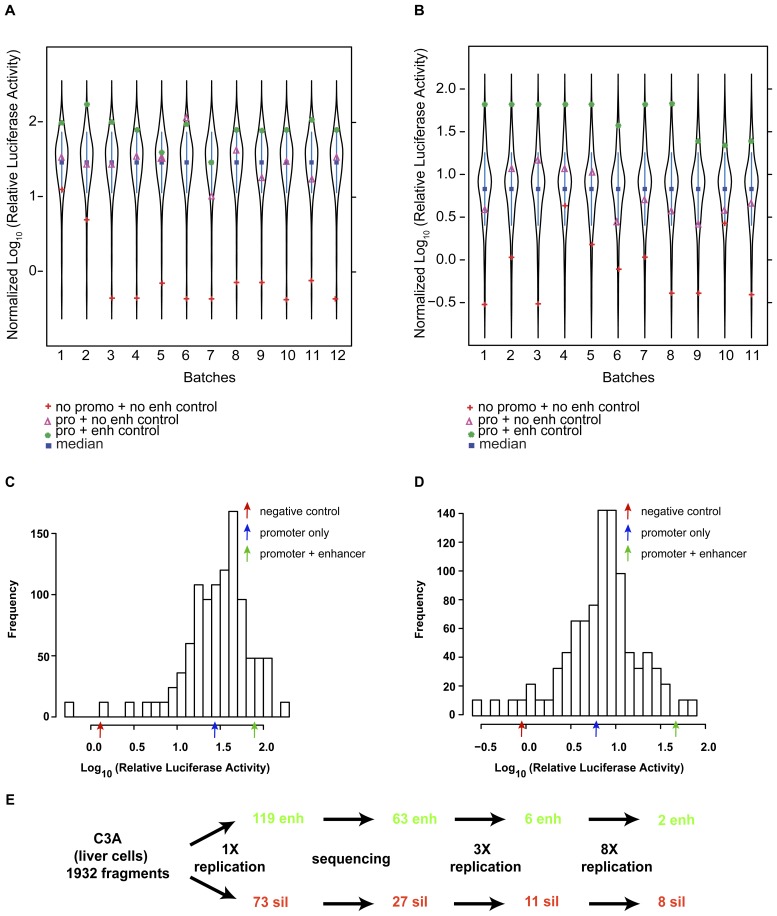
Distributions and workflow. (**A**) Quantile normalized relative luciferase activity for Sau3AI-digested exonic fragments in liver C3A cells compared within, and between, plates. Relative luciferase activity is the log_10_ ratio of firefly luciferase to *Renilla* luciferase. Batch number indicates corresponding 96-well plate. (**B**) Quantile normalized relative luciferase activity for AluI-digested exonic fragments in C3A liver cells. (**C**) Distribution of relative luciferase activities for Sau3aI-digested fragments in liver C3A cells. (**D**) Distribution of relative luciferase activities for AluI-digested fragments in liver C3A cells. (**E**) Workflow for identifying regulatory elements.

Quantile normalization was used to compare luciferase activities across plates, and the activities of clones produced by Sau3AI and AluI digestion were normalized separately (Figure 1A and Figure 1B). Controls acted as expected: vectors with neither a promoter nor enhancer had low activity, vectors with a promoter but no enhancer had moderate activity, and vectors with both a promoter and the known liver enhancer element HCR1 [19] had high activity. The distribution of non-normalized relative luciferase activities was nearly normal and negatively skewed, as described in a previous study [5] (Figure 1C and Figure 1D). The distribution’s unimodality reinforces our previous findings that the distinction between regulatory and nonregulatory sequences is not hard and fast, particularly in the case of enhancers, while the extended negative tail suggests that silencers have a wider range of effect sizes than enhancers [5].

The overall screening procedure was designed to identify coding fragments that reliably show strong regulatory signals, with more stringent thresholds for inclusion at each step (Figure 1E). From the initial, unbiased screen of all 1,932 fragments, we selected additional clones for evaluation with luciferase activity that was two standard deviations beyond the mean. Each of these clones was then sequenced, and its sequence aligned with the human genome (NCBI build 37.2) using BLAT [20]. Non-exonic clones were culled by retaining only those clones whose top BLAT match resided in coding exons, 3′ untranslated regions (UTRs) or 5′ UTRS (all matches had 100% identity, except one unusually long 305 bp fragment with 98% identity). Exonic clones were then subjected in C3A liver cells to two subsequent rounds of testing for regulatory activity, the first round consisting of three replicate assays and the second round consisting of eight. Clones were removed from consideration if they did not demonstrate luciferase activity significantly different from the pGL3-promoter control in each round of assays as determined by one-sample t-tests (df = 2, df = 7, for three and eight replicates, respectively) controlled by false discovery rates (FDR<5% used as threshold for inclusion). We were confident that eight replicates would provide a robust signal of regulatory activity, as luciferase signals across replicates were highly correlated (Pearson correlation, r = 0.978, p<10^−300^).

### Putative Regulatory Elements

**Figure 2:**
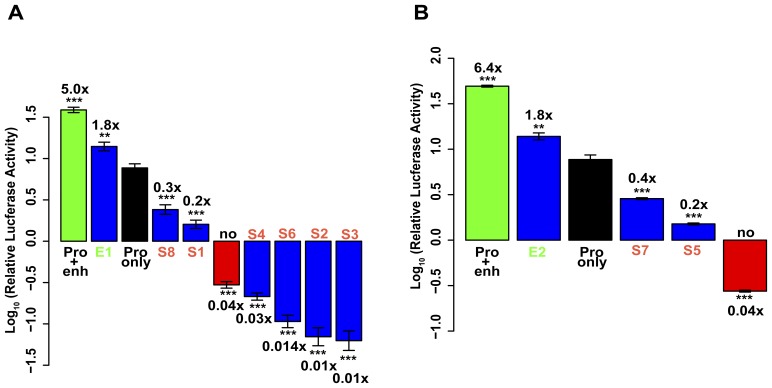
Regulatory activity of putative elements. (**A**) Mean activities of 8 replicates of Sau3AI-digested putative regulatory elements in C3A liver cells. Log_10_ changes relative to promoter-only construct shown. Error bars, standard error of the mean. (**, P<0.01 and ***, P<0.0001 compared to promoter-only construct, both figures.) (**B**) Mean activities of 8 replicates of AluI-digested putative regulatory elements in C3A liver cells.

**Figure 3:**
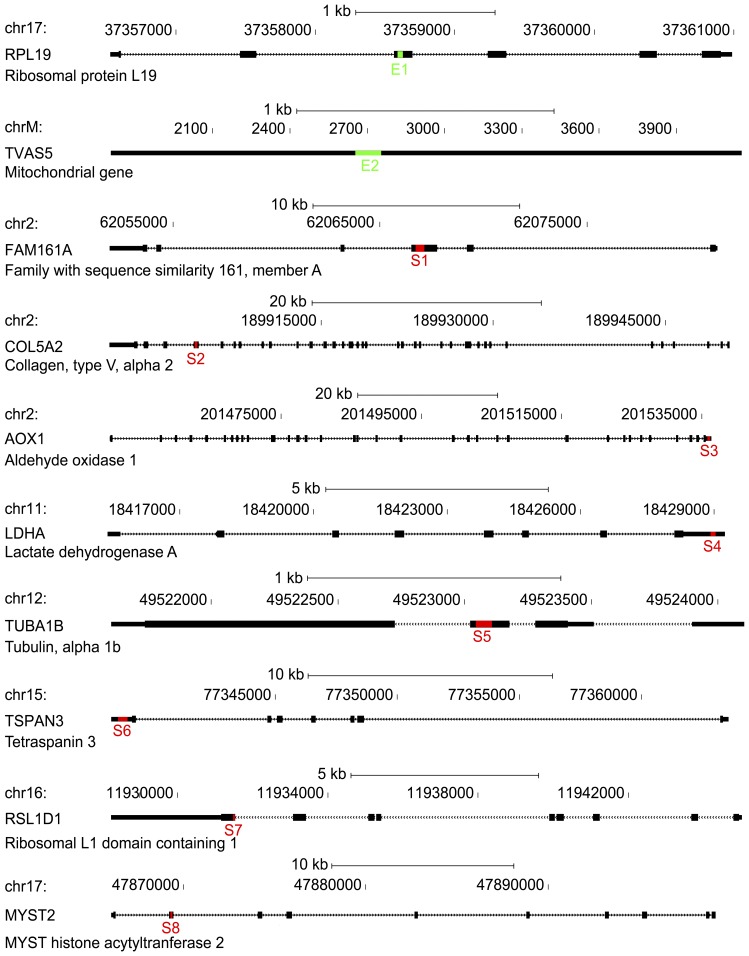
Genomic locations of exonic regulatory elements. Positions of fragments within exons, including coding regions (thick boxes) and 3′ UTRs (thin boxes).

Two clones that showed significantly higher activity than the promoter control across the eight replicates were deemed putative enhancers, while eight clones with lower activity were deemed putative silencers (Figs. 2A and 2B). Genomic locations, lengths and host genes of putative elements are provided in Table S1. Sequences for each element are provided in Table S2. Of the ten putative elements, six resided in coding regions, three in 3′ UTRs, and one resided in the single non-coding exon of a mitochondrial gene (Figure 3).

### Evolutionary Conservation of Putative Regulatory Elements

As judged by phastCons [17] at the UCSC genome browser (http://genome.ucsc.edu/) [21], seven of the ten putative regulatory element sequences were strongly conserved across all vertebrates (mean base-by-base phastCons score for element >0.5), two were somewhat conserved (score >0.1), and one was not conserved at all (score <0.1) (Table S3 and Figure S1). Generally, regulatory elements found in coding exons were more highly conserved than those elements found in 3′ UTRs (mean coding exon score = 0.707±0.143, mean 3′UTR score = 0.392±0.196). As a whole, regulatory element conservation scores reflect both amino acid sequence and regulatory function [8].

To evaluate whether the exonic regulatory elements tended to be more highly conserved than the rest of the host gene, we compared the phastCons conservation score of each regulatory element to the scores of the other exon sequences within the same gene. We used a sampling approach to obtain the null distribution combined with the Wilcoxon-rank sum test (Materials and Methods). Seven of the regulatory elements were significantly more conserved than the exon sequences of their host genes (FDR<0.05), two of the elements (S1 and S3) were significantly less conserved and one element showed no significant difference (Table S3). Fragment S3 may have been significantly less conserved because it resides in an untranslated region. Overall there was a suggestive but inconsistent trend toward increased evolutionary conservation for the regulatory elements, suggesting that both conserved and diverged gene regions can evolve additional transcriptional regulatory functions.

### Sequence Diversity of Putative Regulatory Elements

The inconsistent pattern of increased evolutionary conservation in the exonic regulatory elements may reflect multiple selection pressures on exons, even in the face of additional selection for transcriptional control. We therefore used the statistical power of single nucleotide polymorphism (SNP) data collected from the 1000 Genomes Project (http://www.1000genomes.org/) [22] to ask whether the exonic regulatory elements showed increased conservation in a human population. Using SNPs from 1,094 individuals with a minor allele frequency >1%, we compared the substitution rates for the regulatory elements to all other exonic sequences within the same host gene. The nucleotide substitution rates for the regulatory elements (median rate = 0 bp^−1^) were less than for non-regulatory exon sequences (median rate = 0.0031506 bp^−1^) (Table S4 and Figure S2), with a near suggestive P value (Wilcoxon signed rank test, W = 30, N_r_ = 8, P = 0.1069).

Interestingly, most of the decreased nucleotide divergence in the coding region regulatory elements was due to non-synonymous substitutions (median rates = 0 bp^−1^ and 0.0038306 bp^−1^ in regulatory elements and host gene coding sequences respectively, Wilcoxon signed rank test, W = 10, N_r_ = 4, P = 0.1003). In contrast, the synonymous substitution rate showed diminished differences between the regulatory elements and host gene coding sequences (median rates = 0 bp^−1^ and 0.001497792 bp^−1^ respectively, Wilcoxon signed rank test, W = 10, N_r_ = 5, P = 0.5896). Overall, the trend towards decreased nucleotide substitution rates may imply a selective pressure for coding region regulatory elements in addition to translation alone. A larger database of exonic regulatory elements might further substantiate the notion that these elements possess decreased population diversity.

### Transcription Factor Binding Sites within Putative Regulatory Sequences

We searched for TFBSs using the UCSC Genome Browser ENCODE/HAIB Transcription Factor Binding Sites “peaks” track, which annotates sites with the best evidence (p<10^−5^) for TFBS along the entire human genome as determined by ChIP-seq. Because most DNA-protein interactions were tested in HepG2 liver cells, we confined our search to that cell line. HepG2 cells and the C3A cells used in our study both originate from liver. Transcription factor binding site peaks found within the putative element sequences are listed in Table S5. Four of our putative elements had known TFBSs as determined by ChIP-seq, two with multiple sites. The most common binding site was for HNF4A, hepatocyte nuclear factor 4α. HNF4A is known to be liver-enriched and to target at least 260 genes, possibly thousands of genes covering a wide array of functions [23].

We also employed the UCSC Genome Browser HMR Conserved Transcription Factor Binding Sites track, which uses comparative genomics to predict TFBS locations conserved (p<0.01) across human, mouse, and rat. TFBSs are computationally determined, but not experimentally verified, using TFBS sequence data in the Transfac Matrix Database [14]. Three of our putative silencers contained conserved TFBSs (Figure S3). The S5 silencer located within the coding region of the TUBA1B gene (α-tubulin, 1b) contained two overlapping TFBS for RORA (retinoic acid receptor-related orphan receptor A isoform 1) (z = 2.45, p = 0.0071) and PPARA (peroxisome proliferator-activated receptor α) (z = 2.64, p = 0.0041).

No ChIP-seq data were available for RORA and PPARA from the ENCODE/HAIB Transcription Factor Binding sites “peaks” track. We therefore sought corroborating evidence from related nuclear hormone receptor proteins. ChIP-seq data from ENCODE indicated that the retinoid X receptor, α (RXRA), showed binding to the S5 element (Figure S4A) [24]. In addition, the RXRA TF is known to form a heterodimer with PPARA. RXRA/PPARA heterodimers and RORA monomers bind a common DNA recognition site [24]. Additional evidence for the potential binding of RXRA at the same position on the S5 element as RORA and PPARA was provided by the UniPROBE database, which employs protein binding microarrays to determine transcription factor binding sites on synthetic 8 to 12-mer DNA sequences covering the entire human, mouse and yeast genomes (Figure S4B) [25], [26]. Enrichment scores are calculated by the Wilcoxon-Mann-Whitney test, with a score of 0.5 considered as most favored and −0.5 most disfavored. The significant enrichment score from the UniPROBE database for binding of RXRA to the S5 element was 0.460 [27].

An interplay between retinoic acid receptors and the S5 element was further supported by a significant UniPROBE enrichment score of 0.457 [27] for binding of the retinoic acid receptor, α(RARA) (Figure S6B). RARA and RXRA form a heterodimer (RXRA/RARA) which binds to the same sequence as RXRA/PPARA heterodimers and RORA monomers [24]. It is noteworthy that all four genes, RXRA, RARA, PPARA and RORA are involved in lipid metabolism [28]. It was reported previously that RXRA and RARA act in antagonistic fashion to regulate downstream genes [29]. The analogous binding potential of RXRA and RARA to the same S5 element as RORA and PPARA suggests that RORA and PPARA may also regulate the TUBA1B gene in antagonistic fashion. We explore this potential interaction in more detail below.

### DNase I Hypersensitivity of Putative Regulatory Elements

To investigate whether our putative regulatory elements displayed evidence of open chromatin, we used the UCSC Genome Browser ENCODE/DNase I hypersensitivity (HS) “peaks” track. This track displays DNase I HS across the human genome (p<0.05) in various cell lines as determined by sequencing of DNase I digested genomic DNA. Data from three cell lines, GM12878 (lymphoblastoid), K562 (leukemia) and HepG2 (liver, similar to the C3A liver cell line), were used. DNase I hypersensitivity sites within putative element sequences and their flanking sequences are shown in Figure S5. Signal intensity was estimated using a kernel density estimation function from uniquely mapped DNase I HS tags [30], [31]. The E1 enhancer and all silencer elements had significant signal intensity (p<0.05). No DNase I HS data was available for the E2 element. None of the putative elements had higher DNase I HS signal intensity than their corresponding host gene transcription start site. Overall, it seems that exonic regulatory elements are indeed made available to the transcriptional machinery, but are less open than traditional HS locations.

### GC-content of Putative Regulatory Sequences

GC-rich CpG islands (CGIs) are most often found in the core promoter region immediately upstream of the transcription start site. However, the discovery of a CGI in the intron of the PAX6 gene that may act as an alternative transcription start site has introduced the notion that CGIs not associated with the core promoter may also play a role in transcriptional regulation [32]. To determine whether the putative exonic regulatory elements were found within CGIs, we employed the USCS Genome Browser CpG island prediction track, which identifies sequences at least 200 bp long consisting of >50% GC-content arranged as CpG dinucleotides at least 60% as frequently as expected from GC-content. None of the putative elements were found in CGIs.

It is possible that regulatory elements are actually less likely to be in CGIs than other expressed sequences of the host gene. We therefore compared the GC-content of each element to the GC-content of all exons within the same gene using a sampling strategy combined with the Wilcoxon-rank-sum test (Materials and Methods). One of the ten elements had significantly higher GC-content than their neighboring exons and two of the ten elements had significantly lower GC-content than their neighboring exons at FDR<5% (Table S6). The significantly lower GC content of element S3 was not unexpected, since this element is located within a 3′ UTR and non-coding exons typically have low GC content. As a whole there were no clear patterns of GC content for the exonic regulatory elements, suggesting GC content can be used to predict promoter regions only.

### Histone Modification Signatures of Regulatory Elements

Well-studied histone modifications associated with enhancers include H3K4me1 [15], [16], H3K4me2 [16], H3K27ac [15] and H2A.Z [15]. Histone modifications that predict silencers are not as well known, but several combinations of modifications at promoters have been found to be correlated with low expression, most of which contain H3K27me3 [16]. To determine whether the putative exonic regulatory elements were associated with histone modifications, we aligned our regulatory sequences with the ENCODE/Broad Histone Modification track of the UCSC genome browser, which maps histone modifications across the genome as determined by ChIP-seq across several cell lines including liver. Because only a portion of tested histone modifications were mapped in HepG2 liver cells, we used tracks from all cell types.

Consistent with their diverse contexts, fragments varied in the number and types of histone modifications with which they were associated (Table 1). The E1 enhancer was associated with all 3 known enhancer modifications, H3K4me1, H3K4me2, and H3K27ac, the latter two in liver. On the other hand, four of eight silencers were also associated with H3K4me1 and H3K27ac, although none of them in liver. Repressive signature H3K27me3 was associated with 7 of 8 silencers as well as enhancer E1. Other modifications associated with a majority of fragments include H3K79me2 and H3K20me1.

**Table 1:** Histone modifications associated with fragment sequences^a^.

	H2A.Z	H3K4me1	H3K4me2	H3K27ac	H3K27me3	H3K79me2	H3K20me1
**E1 (RPL19)**	other	other	liver	liver	other	liver	liver
**E2 (TVAS5)** b	–	–	–	–	–	–	–
**S1 (FAM161A)**	other	–	–	–	other	–	–
**S2 (COL5A2)**	liver	–	–	other	other	other	–
**S3 (AOX1)**	other	–	–	–	other	–	other
**S4 (LDHA)**	–	other	–	other	–	–	liver
**S5 (TUBA1B)**	other	other	liver	other	other	liver	liver
**S6 (TSPAN3)**	–	–	–	–	other	liver	liver
**S7 (RSL1D1)**	–	other	–	other	other	liver	liver
**S8 (MYST2)**	–	other	other	other	liver	liver	liver

a “liver” signifies histone modifications associated with fragment in HepG2 cells; “other” signifies histone modifications in cell types other than HepG2.

b No histone modification data for mitochondrial DNA.

### Chromatin State of the Exonic Regulatory Elements

To investigate the enrichment of chromatin states (epigenetic marks) in our putative regulatory elements, we used the UCSC Genome Browser ENCODE Broad ChromHMM track [33], [34]. This track displays chromatin states across the human genome in nine different cell lines by integrating multiple datasets, including ChIP-seq data of histone modifications. The advantage of using ChromHMM, in place of single histone modification data, is that the track summarizes many epigenetic factors.

We found that the regulatory elements varied in their chromatin states (Figures S6 and S7). The E1 enhancer element was found to be enriched with a strong enhancer signal that correlated well with our analysis of enhancer histone signatures (described above). The E2 element had no data in the ChromHMM track. The silencer elements S1, S2 and S3 were enriched in chromatin states suggestive of repressive regions that also correlated well with our analysis of repressive histone signatures (described above). Elements S4, S6, S7, and S8 were broadly enriched in the transcriptional transition state in most of the cell lines. The S5 silencer element resides within the coding region of an exon of the TUBA1B gene and exhibits antagonistic *cis-*regulation by the PPARA and RORA transcription factors (above). The S5 element was found to be enriched in strong enhancer chromatin state in some cell lines using the ChromHMM track. Possibly the S5 silencer has a bidirectional effect based on how it interacts with cell-type specific transcription factors. In general, the majority of the putative regulatory elements displayed chromatin states that were appropriately related to their functional roles.

### *Cis*-regulation of Host Genes

If exonic enhancers and silencers *cis*-regulate expression of their host genes, then manipulation of transcription factors that bind to the regulatory element should alter expression of the host gene. To test this hypothesis, we searched the Gene Expression Omnibus (GEO, http://www.ncbi.nlm.nih.gov/geo) for studies in which the relevant transcription factor was perturbed and the expression of the target gene was measured. We focused on the target gene tubulin α1b (TUBA1B) and the putative silencer S5, located inside one of the coding exons of the gene. Two transcription factors, peroxisome proliferator-activated receptor α (PPARA) and retinoic acid receptor-related orphan receptor α (RORA) have overlapping binding sites within the boundaries of the S5 silencer. Both PPARA and RORA are involved in regulation of lipid metabolism and so are both highly expressed in liver [35], [36].

We first analyzed data from a study in which global gene expression was measured using Affymetrix microarrays in wild-type and PPARA-null mice after administration of either the PPARA agonist WY1463 or after fasting (GEO ID: GSE5475) [35]. Fasting is known to induce PPARA expression in small intestine [37]. To ensure that expression changes due to PPARA induction were specific, we also tested for association between PPARA activation and expression of a control gene, tubulin β4, TUBB4. Tubulin β belongs to the same protein family as tubulin α, but has no known PPARA binding site.

**Figure 4:**
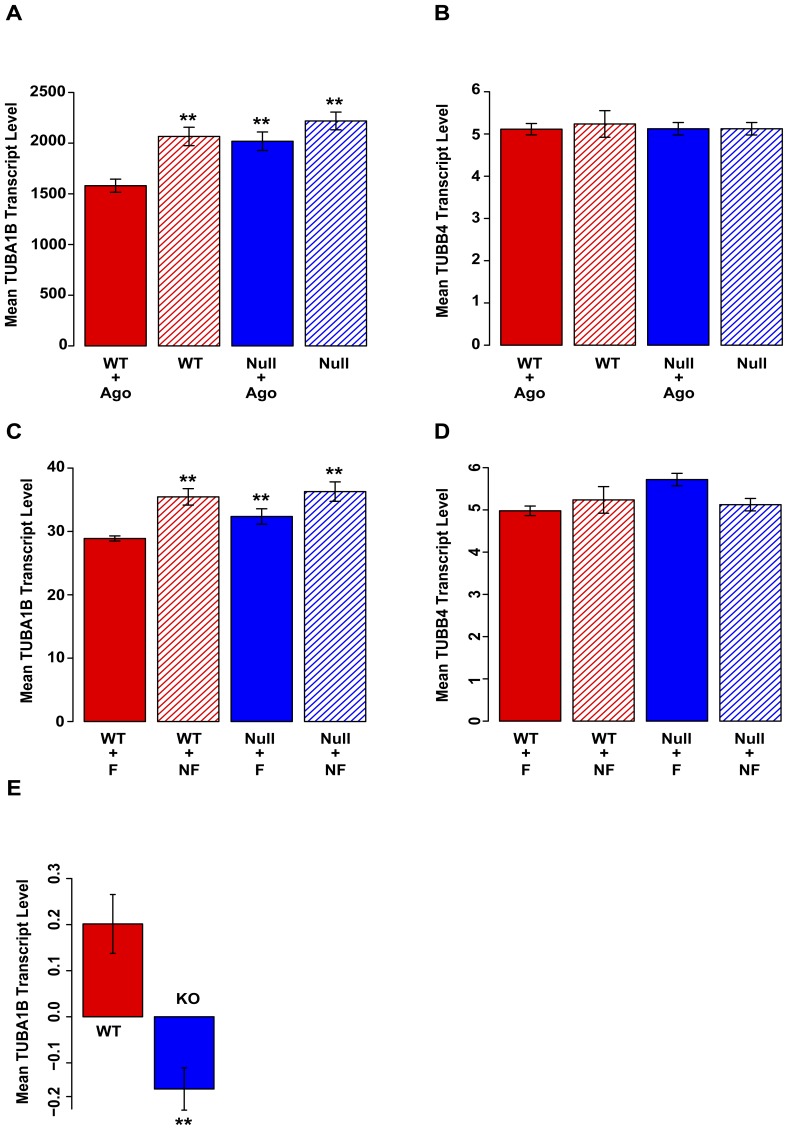
PPARA and RORA regulate TUBA1B expression. For all figures, n = 3 per bar. (**A**) Effects of PPARA genotype (wild-type = red, null = blue) and PPARA agonist WY14643 (present = filled, absent = dashed) on TUBA1B expression in murine small intestine. Error bars, standard error of the mean. (**, P<0.01 compared to wild-type + agonist condition). Only PPARA wild-type mice receiving the PPARA agonist show a reduced TUBA1B expression. Data from [35]. (**B**) PPARA genotype and PPARA agonist do not affect TUBB4 expression. Data from [35]. (**C**) PPARA genotype and fasting (PPARA activation, fasting = filled, no fasting = dashed) effects on TUBA1B expression in murine small intestine (**, p<0.01 compared to wild type + fasting condition). Only PPARA wild-type mice that fasted show reduced TUBA1B expression. Data from [35]. (**D**) PPARA genotype and fasting do not affect TUBB4 expression. Data from [35]. (**E**) RORA activates TUBA1B expression in murine skeletal muscle (**, p<0.01). Data from [36]. All ordinates on log_2_ scale, except (**E**), which is log_10_.

Figure 4A shows the effects of PPARA activation on TUBA1B expression in small intestine in wild type and PPARA-null mice treated with or without the PPARA agonist. Analysis of variance (ANOVA) showed that although there were significant main effects of PPARA-genotype (F = 12.292, df = 1, p = 0.008) and agonist (F = 16.548, df = 1, p = 0.004), the interaction of these two factors was not significant (F = 2.8643, df = 1, p = 0.129). Both main effects appeared to be driven by the decrease in expression in the wild-type/agonist condition compared to the other three conditions (Figure 4A), suggesting that the PPARA-agonist actually affects TUBA1B expression only in wild-type mice. Indeed, post-hoc t-tests indicated that TUBA1B expression is attenuated in wild type mice treated with the PPARA-agonist compared to vehicle treated wild type mice (t = 4.358, df = 4, p = 0.012), but is not attenuated in PPARA-null mice treated with agonist compared to vehicle treated PPARA-null mice (t = 1.583, df = 4, p = 0.189).

In short, the PPARA agonist decreased expression of TUBA1B in wild-type mice, but this decrease was abolished in PPARA-null mice. These results suggest that activation of PPARA decreases expression of the TUBA1B gene and that this decrease is mediated through the PPARA gene. In contrast, ANOVA revealed no significant effects of PPARA-genotype (F = 0.066, df = 1, p = 0.803), PPARA-agonist (F = 0.095, df = 1, p = 0.766), or their interaction (F = 0.095, df = 1, p = 0.7663) on expression of the tubulin β4 (TUBB4) gene (Figure 4B). The absence of an effect on TUBB4 expression as a result of the PPARA agonist is consistent with the lack of a known PPARA binding site in the TUBB4 gene.

Results from the fasting study were similar (Figures 4C and 4D). ANOVA showed that although the fasting effect on TUBA1B expression was significant (F = 19.392, df = 1, p = 0.002), the effects of PPARA-genotype (F = 3.256, df = 1, p = 0.109) and the interaction of fasting and genotype were not (F = 1.228, df = 1, p = 0.3) (Figure 4C). Once again, post-hoc t-tests indicated that fasting wild-type mice had lower expression of TUBA1B than mice who did not fast (t = 4.836, df = 4, p = 0.008), while PPARA-null mice showed no difference when fasting (t = 2.005, df = 4, p = 0.119). Hence fasting, which activates PPARA, causes decreased expression of TUBA1B in wild-type, but not PPARA-null mice, consistent with the notion that TUBA1B expression is repressed via activated PPARA.

In contrast, testing using ANOVA showed that the expression of TUBB4 (Figure 4D) did not depend on PPARA genotype (F = 2.52, df = 1, p = 0.151), fasting (F = 0.727, df = 1, p = 0.419), or their interaction (F = 4.674, df = 1, p = 0.063). This result suggests that TUBB4 is not affected by activated PPARA, again consistent with the lack of a binding site for PPARA in the TUBB4 gene. Together, the results of the pharmacologic and fasting studies suggest that PPARA is a repressor of TUBA1B and that this repression may be mediated by the binding site for PPARA in the exonic silencer S5.

To test for association between RORA activity and TUBA1B expression, we used data from a study using Illumina BeadChip arrays in which global gene expression was compared in skeletal muscle taken from wild-type mice and mice with a RORA dominant negative mutation (GEO ID: GSE20646) [36]. Mean transcript levels of TUBA1B in wild-type and RORA dominant negative mice are shown in Figure 4E. A t-test showed that TUBA1B was expressed at a lower level in RORA dominant negative mice than in wild-type mice (t = 4.5516, df = 4, p = 0.013), suggesting that RORA is an activator of TUBA1B. No data for TUBB4 expression were available from this study [36].

**Figure 5:**
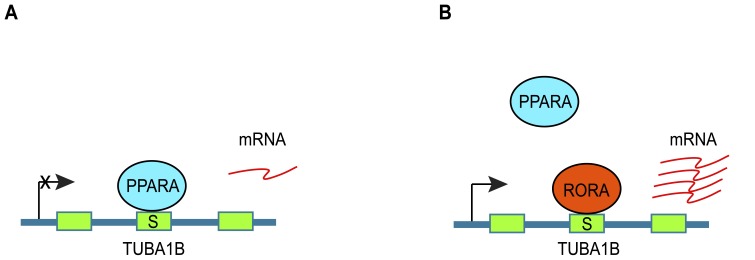
Competition model for PPARA and RORA regulation of TUBA1B expression. (**A**) TUBA1B expression is repressed by binding of PPARA to putative element S5. (**B**) TUBA1B gene expression is activated by displacement of PPARA from S5 by RORA.

From the above observations, we propose that PPARA and RORA compete to bind the TUBA1B regulatory element, wherein PPARA represses TUBA1B when bound, while RORA activates (Figure 5). Consistent with the opposing effects of PPARA and RORA on TUBA1B expression, published reports demonstrate an antagonistic relationship between the two transcription factors. A number of peroxisome proliferated activated receptors, including PPARA and PPARG, as well as orphan nuclear receptors like RORA have highly similar carboxyl terminal extensions in their DNA binding domains that recognize a conserved 5′-extended sequence of some PPAR response elements (PPREs) [24]. PPARs and orphan nuclear receptors compete to bind for overlapping sites, such as those found within the TUBA1B exon. For example, the response element RevDR2, located in the orphan nuclear receptor gene Rev-ErbA, has been shown to mediate repression of its own host gene by Rev-ErbA itself, but to mediate activation by PPARA [24]. Coexpression of Rev-ErbA and PPARA inhibits activation by PPARA [24]. Similarly, RORA and PPARG have overlapping binding sites in the PPRE located in the promoter of the perilipin gene. RORA blocks induction of perilipin through PPARG activation [38].

## Discussion

From a pool of 1,932 random fragments we discovered 10 exonic regulatory elements active in liver within coding regions and 3′ UTRs (as well as a non-coding exon of a mitochondrial gene). A previous screen of 1,798 random fragments from a BAC containing both genic and intergenic DNA from the ApoE gene cluster on chromosome 19 also yielded 10 regulatory elements active in liver [5], suggesting that regulatory elements are as common in exons as they are in the genome as a whole. Since we screened a total of ∼325 kb of transcribed sequences, and there is a total of ∼30 Mb of expressed regions, our work suggests there are at least hundreds of exonic regulatory elements for liver cells in the human genome.

Many of the exonic regulatory elements resided in housekeeping genes, which tend to be expressed at high levels in most cell types. By pooling RNA from a diverse variety of cell types, we attempted to partially normalize the cDNA library and increase transcript coverage. However, the cDNA library was not subtracted, which will cause a bias toward abundant transcripts. The luciferase assay used to screen for the regulatory elements exhibited considerable noise in the initial screening phase of the workflow. However, this variance was mitigated by the use stringent criteria to identify regulatory elements in the follow-up stages, which incorporated replicates of 3× followed by 8×. In addition, selection of each putative element was restricted to a corrected P value with FDR of <5%.

In both our present study of exons and our previous study of the chromosome 19 genome region [5], silencers constituted a substantial portion of the uncovered regulatory elements. Since most assays specifically seek enhancers [39], [40], a large number of regulatory elements may well be missed by current approaches. However, our assay placed the putative silencers upstream of the promoter and it is possible that the uncovered elements may not be functional in their normal coding region location, downstream of the promoter of the gene. For both the uncovered silencers and enhancers, formal proof of function can only come from analysis of the sequences in their natural context.

Nine out of ten of the exonic regulatory fragments were conserved across vertebrates, with seven strongly conserved (phastCons score >0.5). The evolutionary conservation of the regulatory fragment was correlated with conservation of the host gene as a whole, and fragments within coding regions were more conserved than those in 3′ UTRs. Others have shown that computationally predicted exonic regulatory elements have lower nucleotide substitution rates than other coding exons within the same host gene, presumably because of dual selective pressure to preserve both protein-coding sequence and TFBSs [8]. Consistent with lower substitution rates, we found that seven of our regulatory elements were more conserved than other exon sequences of the host gene, whereas two fragments – one of which was coding – were significantly less conserved. Perhaps, then, some exonic regulatory elements are released from selective pressure, possibly as a means to allow for transcriptional control while still preserving protein composition.

Complementing our findings of increased conservation at the evolutionary level, we also showed that the coding region regulatory elements displayed a trend toward decreased polymorphism diversity using data from the 1000 Genomes Project. The decreased diversity of the elements in the human population was largely due to decreased non-synonymous substitution rates. These observations suggest that the coding region regulatory elements are under additional selective pressure than translation alone. The trend towards decreased diversity is also consistent with our findings of increased evolutionary conservation for some of the regulatory elements. Perhaps the exonic elements exert a subtle selective pressure that can best be detected in the context of intraspecies evolution. A larger database of exonic regulatory elements will allow this possibility to be addressed with more certainty.

Traditionally, high GC-content has been associated with core promoter sequences, while thus far evidence of association between distal regulatory elements and higher GC-content is scarce [32]. Most exonic regulatory fragments had GC-content higher than the genome as a whole, but much like conservation, it is difficult to separate whether high GC-content is associated with coding or regulatory function or both. In contrast, we found that no exonic regulatory fragments resided within CpG islands (CGI) and some fragments had lower GC-content than neighboring exons within the same host gene. Currently, it seems GC-content and CGI residence would be best left to predict promoters only.

Six fragments had predicted TFBSs determined by ChIP-seq and comparative genomics. Three fragments had binding sites for HNF4A, hepatocyte nuclear factor 4α. HNF4A is known to be a master regulator of the expression of a wide variety of genes in liver [23], so this finding is unsurprising. Enhancer E2 was found to have five predicted transcription factor binding sites. However, the enhancer resides within the single coding exon of mitochondrial gene TVAS5. While five predicted TFBSs would typically suggest that E2 is a true enhancer, the TVAS5 element is not exposed to the nuclear regulatory environment. Much work would be necessary before it could be accepted that this element possesses a physiological role.

Using comparative genomics, ChIP-seq and genome-wide binding assays, overlapping conserved TFBSs for PPARA, RORA and other nuclear receptors were predicted within S5, a putative silencer in the coding region of TUBA1B. The S5 silencer also mapped to a processed pseudogene in a different region of the genome (chromosome 11; 90016133-90016194) with the same score as TUBA1B. However, unlike the TUBA1B silencer element, the second location did not display any enhancer-like signatures in the UCSC Genome Browser histone modification or ChromHMM ENCODE tracks (not shown), making it less likely that this region acts as a regulatory element.

We verified the *cis*-regulatory potential of the TUBA1B silencer by positively and negatively correlating expression of TUBA1B with RORA and PPARA activation, respectively. Nuclear receptors, like RORA and PPARs, have been shown to have opposing effects on downstream gene expression [24], [38]. One interesting implication of this relationship is that TUBA1B silencer S5 may have been discovered as an enhancer had the complement of TFs in the cell assay been different, for example if RORA were overexpressed relative to PPARA. It is possible many regulatory elements may have bidirectional effects, depending on TF interactions, and what were once known as “enhancers” and “silencers” may be more appropriately called “regulators”.

The exonic regulatory elements exhibited a degree of open chromatin conformation as judged using DNase I HS, but the extent was less than found at traditional transcriptional initiation sites. The versatility and difficulty in identifying regulatory elements is reflected in the diversity of histone modifications associated with them. Most of the signatures previously used to identify enhancers, such as H3K4me1, were associated with several exonic silencers in our work. Because not all histone modifications were mapped in HepG2 liver cells, we also looked at the chromatin state at the position each of our putative exonic regulatory elements in all other cell types tested for the UCSC Genome Browser ENCODE/Broad Histone Modification track. Cross-cell type inferences should be made cautiously, as histone modifications at enhancers are known to vary considerably between cell types [15]. Nevertheless, no modification clearly delineated the boundary between regulatory and non-regulatory exon fragments, or between enhancer and silencer. Indeed, histone modifications often correlate with each other, suggesting that rather than individual modifications, modules consisting of many interacting modifications are the true markers of regulatory activity [16].

To overcome the limitations of examining any one histone modification in isolation, we turned to the UCSC Genome Browser ENCODE Broad ChromHMM track. This track improves the reliability of chromatin assignments by integrating multiple data types, including histone ChIP-seq datasets. In general, we found reasonable agreement between the chromatin states and functions of the exonic elements. However, the function of the exonic regulatory elements can potentially change in various cellular contexts. One strategy to investigate this possibility would be to test the putative elements in different cell lines.

Exonic regulatory elements appear to be sequences that are concurrently conserved, enriched in TFBSs and associated with several histone modifications. This likely reflects the biology: a given TFBS motif appears many times in the genome, and only a fraction are likely true binding sites, most likely those which are clustered together, in which access to the TFBSs is permitted by histone modifications and those where the TFBSs are conserved across species. No feature correlates perfectly with regulatory activity, so single-feature based approaches are likely to fail. Integrated approaches have already been successfully used to predict the locations of coding regulatory elements. For example, a search for clusters of TFBS conserved spatially and evolutionary across human, mouse, and rat was used to predict ∼700,000 regulatory elements, including an experimentally-verified coding enhancer for the gene ADAM metallopeptidase with thrombospondin type 1 motif, 5 **(**ADAMTS5) [7].

In sum, the variability of coding regulatory elements requires that genome-wide searches will have to define an appropriately multifaceted signature. The complexity of interactions between DNA, transcription factors and chromatin state should be integrated into tools used to search for the sequences where these interactions occur. Nevertheless, the bioinformatic identification of regulatory elements, both exonic and non-exonic, will clearly benefit from the development of rapid experimental tools that can permit direct genome-wide evaluation of transcriptional control sequences rather than depending upon related, but ultimately, surrogate markers of regulation.

## Materials and Methods

### Cell Culture and cDNA Synthesis

To normalize transcript levels used to generate cDNA, RNA was pooled in equal amounts from three human cell lines; HEK-293 (kidney), C3A (liver) and SvGp12 (astrocyte) (all from ATCC). Cell lines were grown in Eagle’s Minimum Essential medium (ATCC 30-2003) and 10% fetal bovine serum (Invitrogen) until 75% confluency was reached. For each cell line, the Oligotex mRNA mini kit (Qiagen) was used to isolate and purify mRNA. Pooled mRNA from all cell lines then used to synthesize cDNA using the Just cDNA Double Stranded cDNA Synthesis Kit (Agilent). Random hexamers were chosen as primers to avoid the 3′ bias of oligo-dT primers. Quality of RNA and cDNA were assessed using spectrophotometry and gel electrophoresis, respectively.

### Library Construction

Samples of pooled cDNA were digested by either Sau3AI or AluI (New England Biolabs) and sub-cloned into the pGL3-promoter vector (Promega), digested with SmaI or BglII, respectively. Vectors were then transformed into MAX efficiency DH5α chemically competent bacteria (Invitrogen), clones isolated, and plasmid DNA purified using 96 Plasmid Miniprep Kit (Qiagen).

### Control Clones

The pGL3-promoter and pGL3-basic vectors, both from Promega, served as neutral (promoter, but neither enhancer nor silencer) and negative controls (no promoter, enhancer or silencer), respectively. The reporter gene for both vectors was firefly luciferase. For a positive control, we used the previously identified human APOE liver-specific enhancer HCR1 inserted into the pGL3- promoter vector [19].

### Transfection and Reporter Gene Activity Assays

For each clone, 100 ng of firefly experimental luciferase plasmid and 10 ng of control *Renilla* luciferase plasmid (phRL-TK, Promega) were co-transfected into C3A human liver cells (ATCC) using the Effectene transfection reagent (Qiagen) in 96-well plates. The *Renilla* plasmid serves as a control for transfection efficiency. Transfection was performed when cells had reached 80% confluency. Cells were then grown in Eagle’s Minimum Essential medium (ATCC 30-2003) and lysed after 24 hours. Luciferase reporter gene activity was assayed using the Dual-Luciferase Assay Kit (Promega).

### Screens and Sequencing

Relative luciferase activity, the log_10_ ratio of firefly to *Renilla* luciferase signal, was used as a measure of expression relative to transfection efficiency. Raw activity ratios were quantile normalized across 96-well plates. Clones were chosen for further screening upon demonstrating activity two standard deviations away from the mean after normalization. Sequencing of putative clones was performed at GenoSeq, the UCLA genotyping and sequencing core.

### Testing PhastCons and GC Content Scores of the Regulatory Elements

For each host gene, a sliding window of length equal to the regulatory element length was employed to obtain phastCons conservation and GC content scores from the UCSC genome browser. The window was shifted in single base pair increments along the entire length of the expressed region of the host gene to obtain a null distribution of score values for the gene. The Wilcoxon-rank sum test was then used to compare values of the regulatory elements with the null distributions obtained from the host genes.

## Supporting Information

Figure S1 Conservation of fragments. PhastCons scores, which represent the probability that a base is conserved across vertebrates, for all bases in each fragment sequence.
(TIF)

Figure S2 Nucleotide substitution rates. Comparison of nucleotide substitution rate for exonic regulatory elements and remaining exon sequences of host genes.
(TIF)

Figure S3 Conserved transcription factor binding sites. Positions of transcription factor binding sites conserved across human, mouse and rat relative to amino acid sequence of fragment.
(TIF)

Figure S4 RXRA and RARA transcription factor binding sites on S5 element. (**A**) RXRA binding site on S5 element determined by ChIP-seq. (**B**) RXRA and RARA binding sites on S5 element determined by protein binding microarray data from UniPROBE.
(TIF)

Figure S5 DNase I hypersensitive sites. Positions of DNase I hypersensitive sites for the exonic regulatory elements in cell lines GM12878, K562 and HepG2.
(TIF)

Figure S6 Chromatin state of the exonic regulatory elements. Signatures of epigenetic marks (chromatin states) obtained using the ChromHMM ENCODE track for the exonic regulatory elements E1, S1, S2, S3, and S4 in nine cell lines.
(TIF)

Figure S7 Chromatin state of the exonic regulatory elements. Signatures of epigenetic marks (chromatin states) obtained using the ChromHMM ENCODE track for the exonic regulatory elements S5, S6, S7 and S8 in nine cell lines.
(TIF)

Table S1 Putative regulatory elements.
(DOC)

Table S2 Fragment sequences.
(DOC)

Table S3 Conservation of regulatory elements and host exon sequences.
(DOC)

Table S4 Nucleotide substitution rate of regulatory elements.
(DOC)

Table S5 Transcription factor binding sites determined by ChIP-seq.
(DOC)

Table S6 GC content of regulatory elements and host exon sequences.
(DOC)
