# Supplementary figures and images for: Discovery and Characterization of Human Exonic Transcriptional Regulatory Elements
Source: PLoS One. 2012 Sep 24;7(9):e46098. doi: 10.1371/journal.pone.0046098 (PMC3454335; doi:10.1371/journal.pone.0046098)

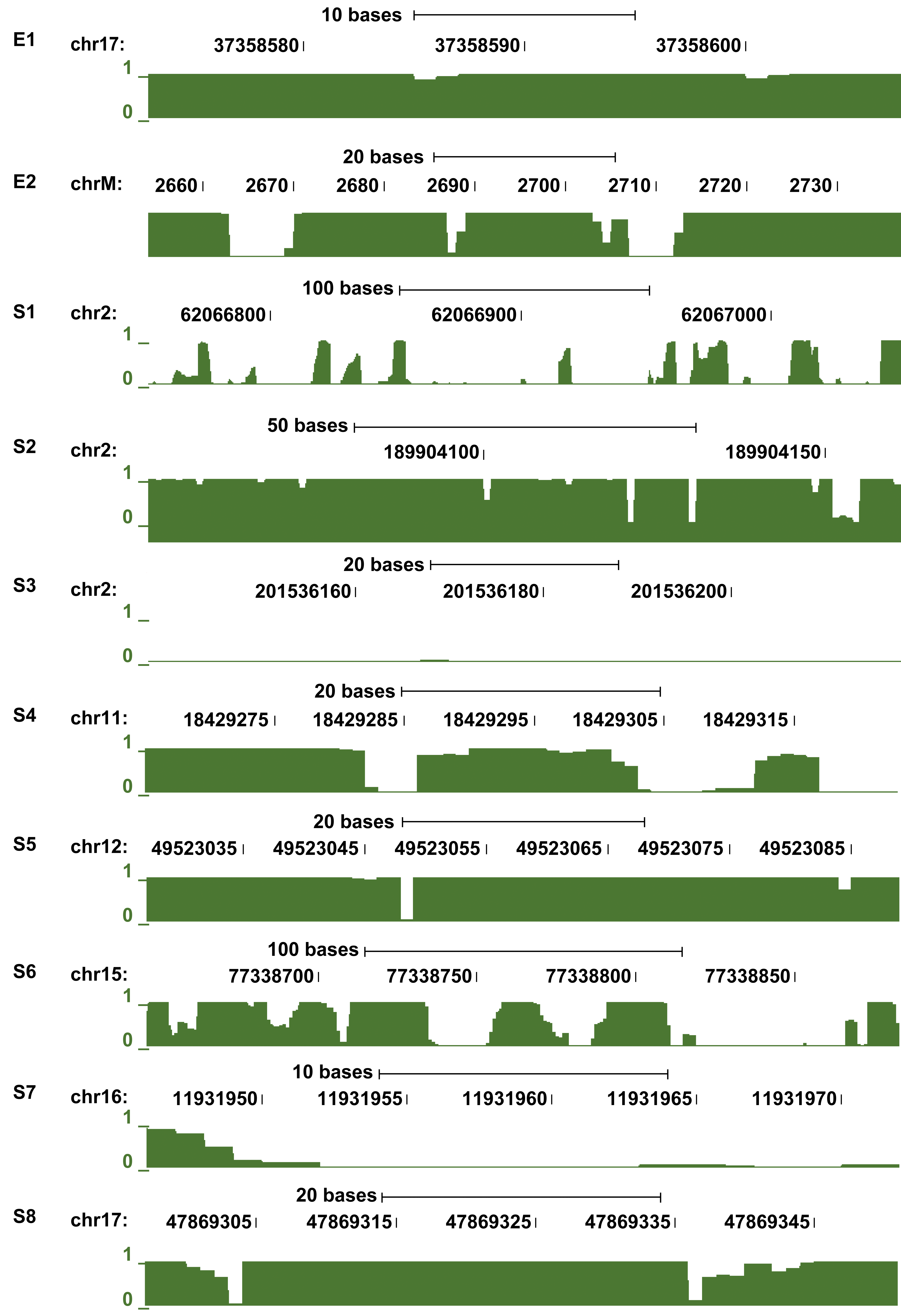

Supplement: Figure S1 — Conservation of fragments. PhastCons scores, which represent the probability that a base is conserved across vertebrates, for all bases in each fragment sequence. (TIF) [file pone.0046098.s001.tif]

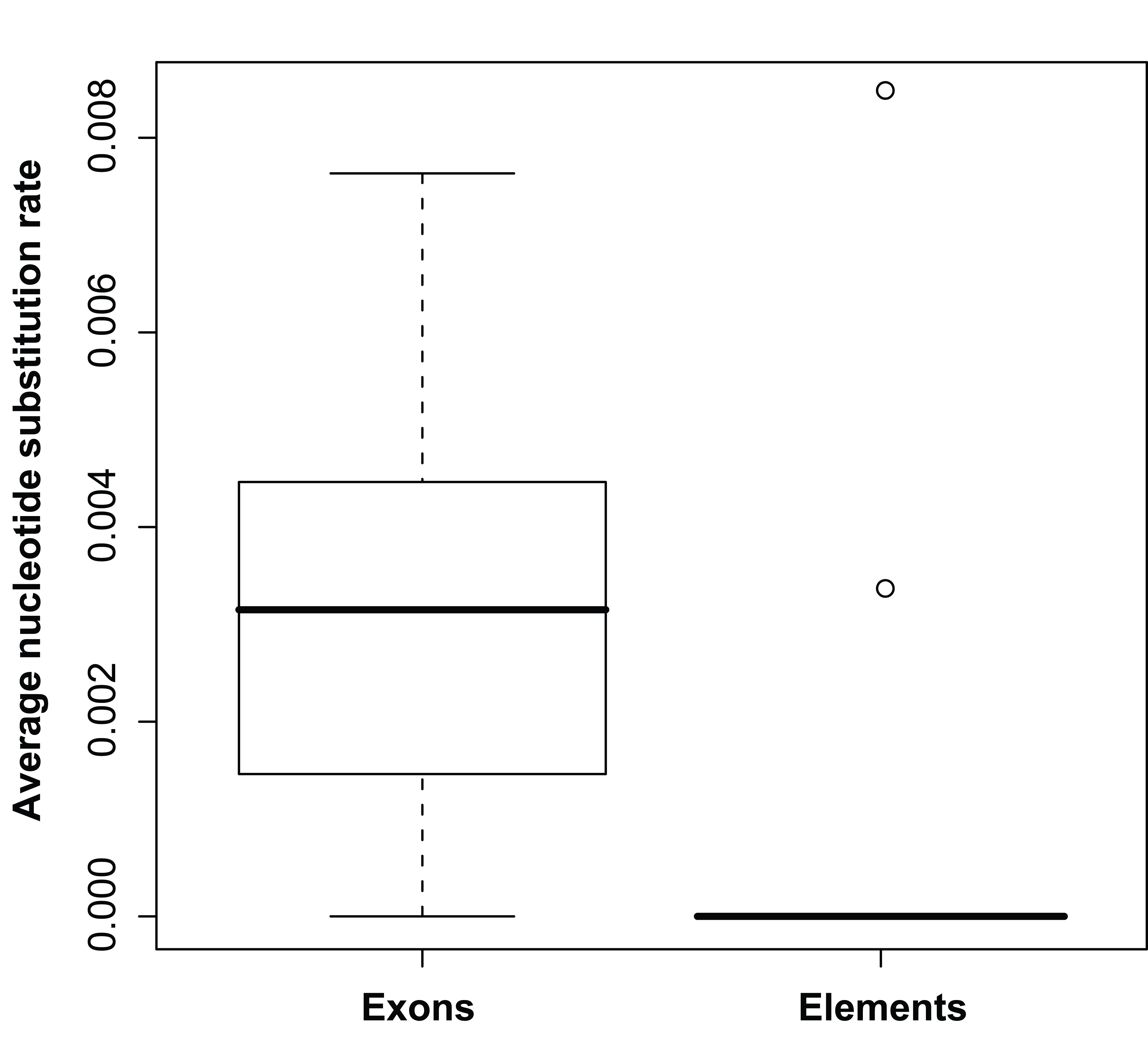

Supplement: Figure S2 — Nucleotide substitution rates. Comparison of nucleotide substitution rate for exonic regulatory elements and remaining exon sequences of host genes. (TIF) [file pone.0046098.s002.tif]

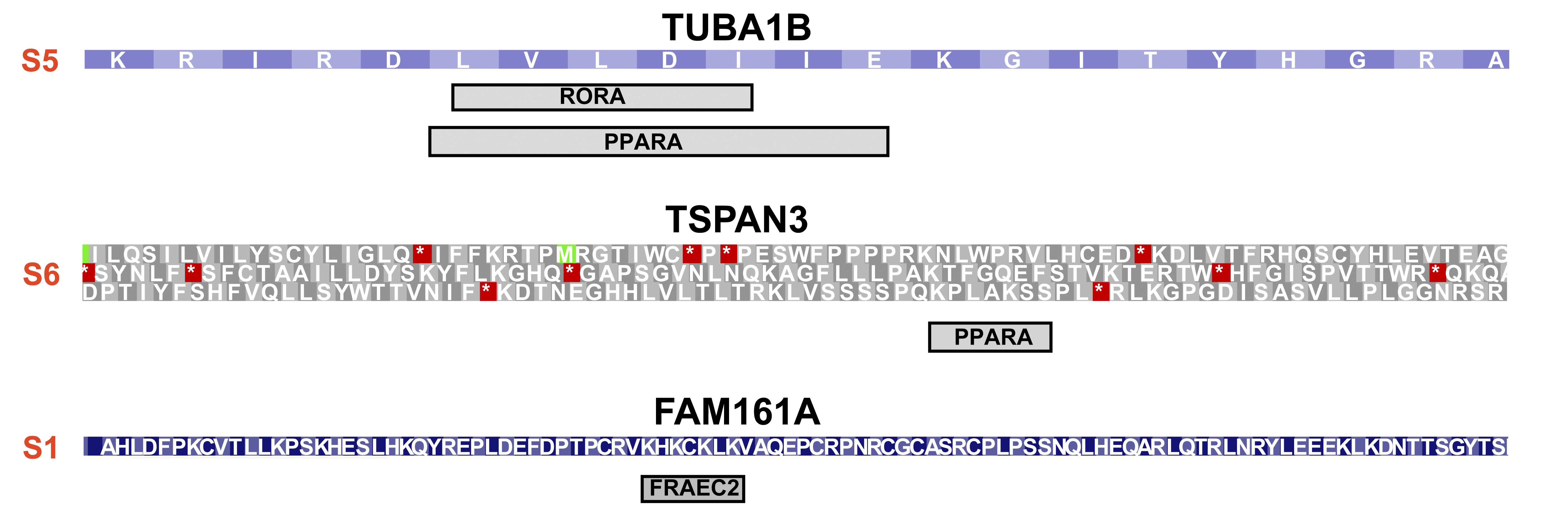

Supplement: Figure S3 — Conserved transcription factor binding sites. Positions of transcription factor binding sites conserved across human, mouse and rat relative to amino acid sequence of fragment. (TIF) [file pone.0046098.s003.tif]

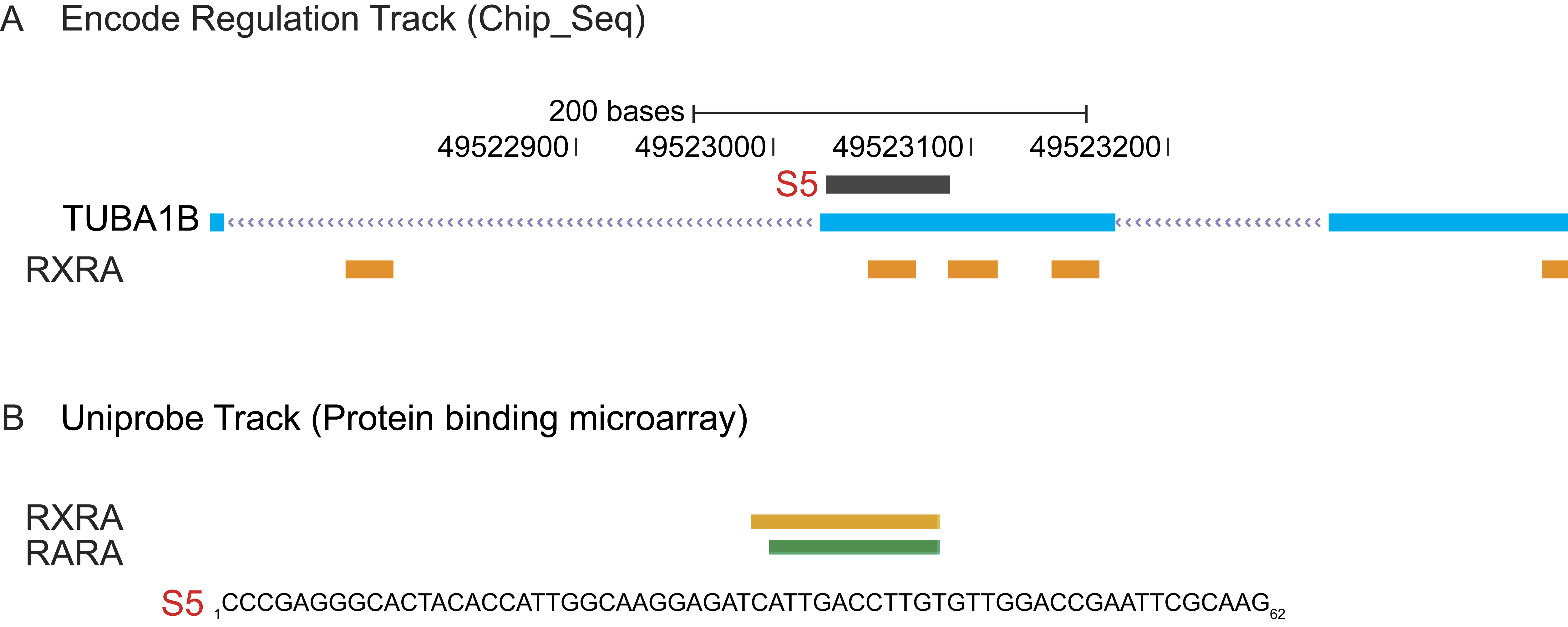

Supplement: Figure S4 — RXRA and RARA transcription factor binding sites on S5 element. (A) RXRA binding site on S5 element determined by ChIP-seq. (B) RXRA and RARA binding sites on S5 element determined by protein binding microarray data from UniPROBE. (TIF) [file pone.0046098.s004.tif]

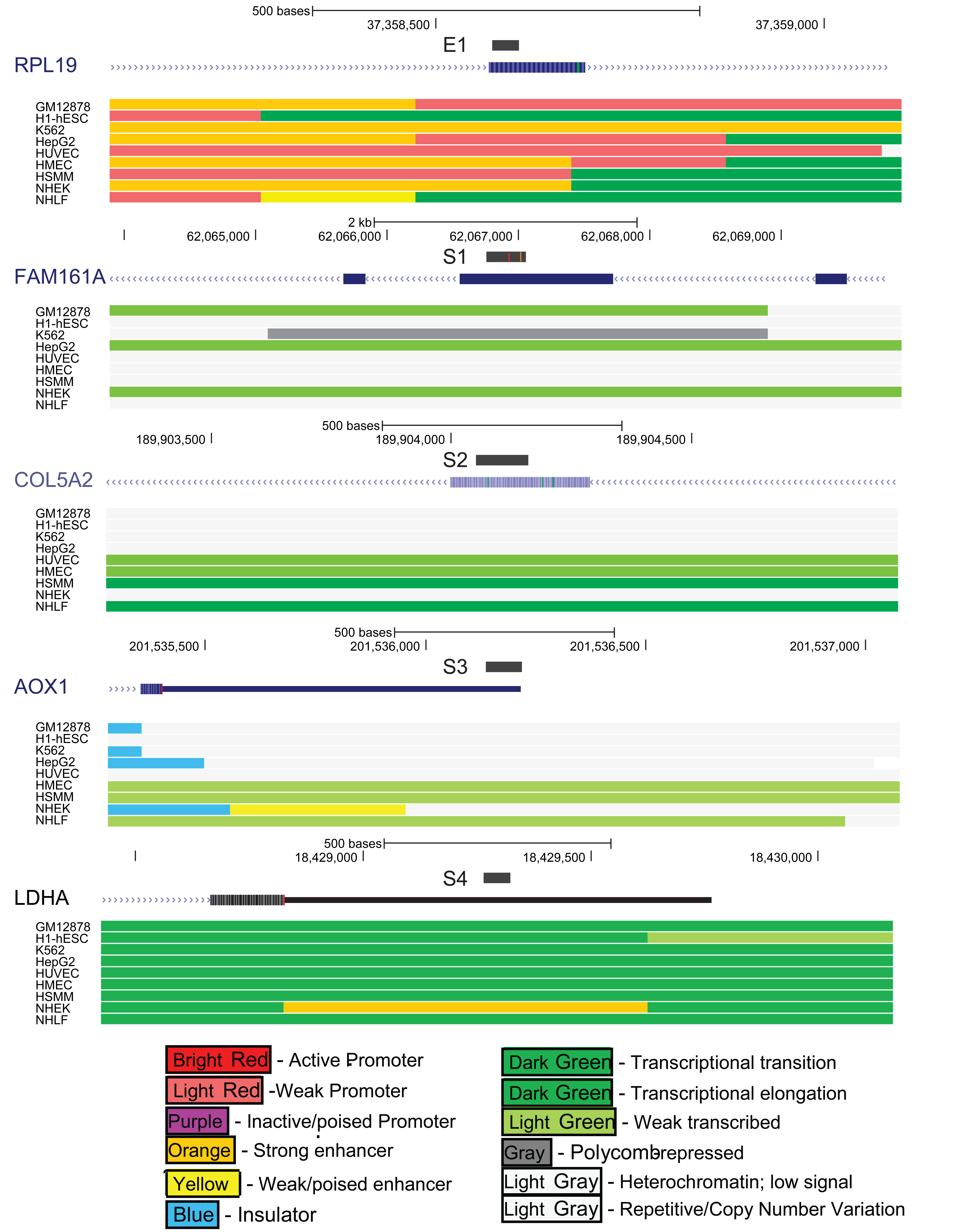

Supplement: Figure S6 — Chromatin state of the exonic regulatory elements. Signatures of epigenetic marks (chromatin states) obtained using the ChromHMM ENCODE track for the exonic regulatory elements E1, S1, S2, S3, and S4 in nine cell lines. (TIF) [file pone.0046098.s006.tif]

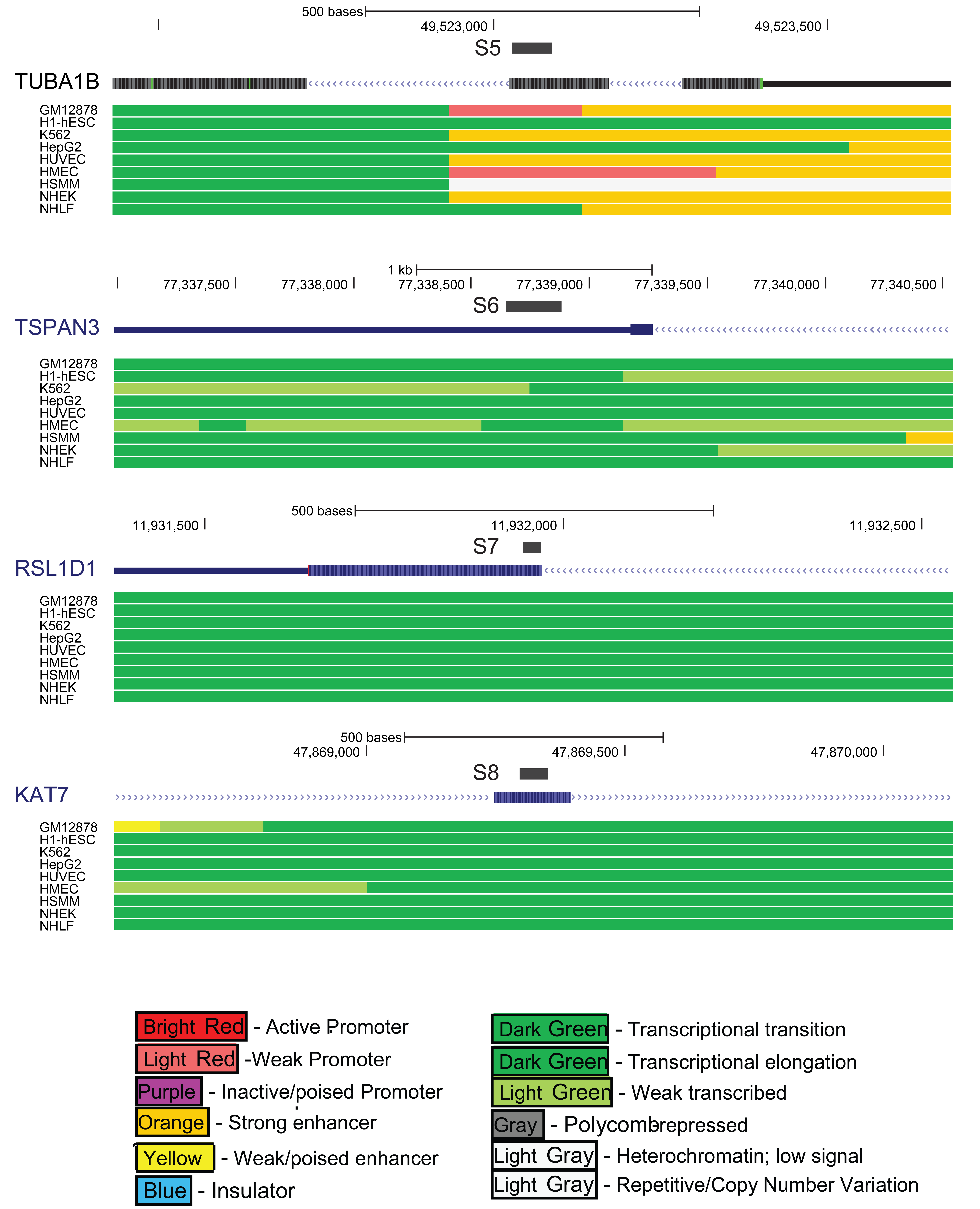

Supplement: Figure S7 — Chromatin state of the exonic regulatory elements. Signatures of epigenetic marks (chromatin states) obtained using the ChromHMM ENCODE track for the exonic regulatory elements S5, S6, S7 and S8 in nine cell lines. (TIF) [file pone.0046098.s007.tif]
