# Supplementary material for: Discovery and Characterization of Human Exonic Transcriptional Regulatory Elements
Source: PLoS One. 2012 Sep 24;7(9):e46098. doi: 10.1371/journal.pone.0046098 (PMC3454335; doi:10.1371/journal.pone.0046098)
Supplement: Table S1 — Putative regulatory elements. (DOC) [file pone.0046098.s008.doc]

**Table S1. Putative regulatory elements.**

| **Element^a^** | **Restriction enzyme** | **Gene** | **Region** | **Start position** | **Length (bp)** |
| --- | --- | --- | --- | --- | --- |
| **E1** | Sau3AI | RPL19 | Coding | chr17:37,358,574 | 34 |
| **E2** | AluI | TVAS5 | Coding | chrM:2,655 | 83 |
| **S1** | Sau3AI | FAM161A | Coding | chr2:62,066,752 | 305 |
| **S2** | Sau3AI | COL5A2 | Coding | chr2:189,904,052 | 110 |
| **S3** | Sau3AI | AOX1 | 3'UTR | chr2:201,536,139 | 80 |
| **S4** | Sau3AI | LDHA | 3'UTR | chr11:18,429,266 | 58 |
| **S5** | AluI | TUBA1B | Coding | chr12:49,523,028 | 62 |
| **S6** | Sau3AI | TSPAN3 | 3'UTR | chr15:77,338,647 | 237 |
| **S7** | AluI | RSL1D1 | Coding | chr16:11,931,947 | 26 |
| **S8** | Sau3AI | MYST2 | Coding | chr17:47,869,298 | 54 |

^a^ Elements labeled “E” are putative enhancers; elements labeled “S” are putative silencers
