# Supplementary material for: Discovery and Characterization of Human Exonic Transcriptional Regulatory Elements
Source: PLoS One. 2012 Sep 24;7(9):e46098. doi: 10.1371/journal.pone.0046098 (PMC3454335; doi:10.1371/journal.pone.0046098)
Supplement: Table S2 — Fragment sequences. (DOC) [file pone.0046098.s009.doc]

**Table S2. Fragment sequences.**

| **Element** | **Sequence** |
| --- | --- |
| **E1** | GATCAGCCCATCTTTGATGAGCTTCCGGATCTGC |
| **E2** | CTGTCTCTTACTTTTAACCAGTGAAATTGACCTGCCCGTGAAGAGGCGGGCATGACACAGCAAGACGAGAAGACCCTATGGAG |
| **S1** | TCTACTTATGGTTCAACTACCAATGACAAGTTAAAAGAAGAAGAAGCTCTATCGAAACCTTAGGACACAGCTGAGAGCCCAGGAGCATTTACAGAACTCATCTCCTCTGCCTTGTAGGTCAGCTTGCGGATGCAGGAACCCCAGGTGTCCTGAACAGGCTGTAAAGTTGAAGTGTAAACACAAGGTTAGGTGCCCAACTCCTGATTTTGAGGACCTTCCTGAGAGATACCAGAAACACCTCTCAGAACACAAGTCTCCAAAACTCTTAACAGTGTGTAAACCATTTGATCTGCTGATCTGCATCTC |
| **S2** | TCAGGCGGCTCCTGATGACAAAAACAAAACGGACCCAGGGGTTCATGCTACCCTGAAGTCACTCAGTAGTCAGATTGAAACCATGCGCAGCCCCGATGGCTCGAAAAAGC |
| **S3** | GATCATTTAACATTCTGTGTATGTAACAAAATATCACATGCATAAATATTATGTATCAATAAAATT TTTTAATGGGCAAA |
| **S4** | AGATCTTTTTACATTATATGGTAATGTACACTACTGATATAGTTCACAAAATAAGATC |
| **S5** | CCCGAGGGCACTACACCATTGGCAAGGAGATCATTGACCTTGTGTTGGACCGAATTCGCAAG |
| **S6** | GATCCTACAATCTATTTTAGTCATTTTGTACAGCTGCTATCTTATTGGACTACAGTAAATATTTTTTAAAAGGACACCAATGAGGGGCACCATCTGGTGTTAACCTTAACCAGAAAGCTGGTTTCCTCCTCCTCCCCGCAAAAACCTTTGGCCAAGAGTTCTCCACTGTGAAGACTGAAAGGACCTGGTGACATTTCGGCATCAGTCCTGTTACCACTTGGAGGTAACAGAAGCAGG |
| **S7** | AGATTCAAAAACATGCCACAGGAAAG |
| **S8** | AGATCTCGAGCACACAGACAGTTCAGAAAGTGATGGCACATCCCGACGATCTGC |
