# Supplementary material for: Discovery and Characterization of Human Exonic Transcriptional Regulatory Elements
Source: PLoS One. 2012 Sep 24;7(9):e46098. doi: 10.1371/journal.pone.0046098 (PMC3454335; doi:10.1371/journal.pone.0046098)
Supplement: Table S3 — Conservation of regulatory elements and host exon sequences. (DOC) [file pone.0046098.s010.doc]

Table S3. Conservation of regulatory elements and host exon sequences.

| **Element** | **Mean phastCons score for element** | **Mean phastCons score for all exons** | **Median phastCons score for element** | **Median phastCons score for all exons** | **U** | **P-value** | **FDR** |
| --- | --- | --- | --- | --- | --- | --- | --- |
| **E1** | 0.989 | 0.892 | 1 | 0.977 | 3576 | 1.42E-11 | 2.367E-11 |
| **E2** | 0.820 | 0.562^a^ | 1 | 0.564^a^ | 36168 | 2.20E-16 | 4.4E-16 |
| **S1** | 0.211 | 0.349 | 0 | 0.182 | 678071 | 2.20E-16 | 4.4E-16 |
| **S2** | 0.932 | 0.746 | 1 | 0.924 | 61972.5 | 2.20E-16 | 4.4E-16 |
| **S3** | 0.004 | 0.624 | 0 | 0.712 | 378481 | 2.20E-16 | 4.4E-16 |
| **S4** | 0.532 | 0.631 | 0.854 | 0.585 | 47123 | 0.042 | 0.0470 |
| **S5** | 0.977 | 0.504 | 1 | 0.428 | 10385 | 2.20E-16 | 4.4E-16 |
| **S6** | 0.540 | 0.328 | 0.559 | 0.023 | 314362 | 2.39E-08 | 2.988E-08 |
| **S7** | 0.110 | 0.137 | 0.016 | 0.017 | 80948 | 0.105 | 0.105 |
| **S8** | 0.921 | 0.803 | 1 | 0.972 | 49085 | 6.84E-10 | 9.771E-10 |

^a^ only one exon for this gene
