# Supplementary material for: Discovery and Characterization of Human Exonic Transcriptional Regulatory Elements
Source: PLoS One. 2012 Sep 24;7(9):e46098. doi: 10.1371/journal.pone.0046098 (PMC3454335; doi:10.1371/journal.pone.0046098)
Supplement: Table S4 — Nucleotide substitution rate of regulatory elements. (DOC) [file pone.0046098.s011.doc]

# Table S4. Nucleotide substitution rate of regulatory elements.

| **Element** | **Gene** | **Nucleotide substitution rate of exon sequences (bp^-1^)** | **Nucleotide substitution rate of elements (bp^-1^)** |
| --- | --- | --- | --- |
| **E1** | RPL19 | 0 | 0 |
| **E2** | TVAS5 | NA | NA |
| **S1** | FAM161A | 0.00413101 | 0.003322259 |
| **S2** | COL5A2 | 0.00544806 | 0 |
| **S3** | AOX1 | 0.00185529 | 0 |
| **S4** | LDHA | 0.00146199 | 0 |
| **S5** | TUBA1B | 0.00446286 | 0 |
| **S6** | TSPAN3 | 0.0031506 | 0.00844 |
| **S7** | RSL1D1 | 0.00763359 | 0 |
| **S8** | MYST2 | 0.00146199 | 0 |

Test statistic of nucleotide substitution rate of all element sequences vs. all exon sequences of host genes, W = 30, P-value = 0.1069 (Wilcoxon signed rank test)
