# Supplementary material for: Discovery and Characterization of Human Exonic Transcriptional Regulatory Elements
Source: PLoS One. 2012 Sep 24;7(9):e46098. doi: 10.1371/journal.pone.0046098 (PMC3454335; doi:10.1371/journal.pone.0046098)
Supplement: Table S5 — Transcription factor binding sites determined by ChIP-seq. (DOC) [file pone.0046098.s012.doc]

## Table S5. Transcription factor binding sites determined by ChIP-seq.

| E1 | HNF4A |
| --- | --- |
| E2 | CTCF, HNF4A, p300, YY1, ZBTB33 |
| S4 | FOSL2, FOXA1, HEY1, HNF4A, JunD, SP2 |
| S5 | HEY1 |
