# Supplementary material for: Discovery and Characterization of Human Exonic Transcriptional Regulatory Elements
Source: PLoS One. 2012 Sep 24;7(9):e46098. doi: 10.1371/journal.pone.0046098 (PMC3454335; doi:10.1371/journal.pone.0046098)
Supplement: Table S6 — GC content of regulatory elements and host exon sequences. (DOC) [file pone.0046098.s013.doc]

**Table S6. GC content of regulatory elements and host exon sequences.**

| **Element** | **Mean GC content of element** | **Mean GC content of all exons** | **Median**  **GC content for element** | **Median GC content for all exons** | **U** | **P-value** | **FDR** |
| --- | --- | --- | --- | --- | --- | --- | --- |
| **E1** | 49.738 | 53.712 | 59.843 | 53.881 | 406 | 0.891 | 0.906 |
| **E2** | 49.606 | 44.201 | 39.370 | 43.214 | 3628 | 0.833 | 0.906 |
| **S1** | 44.014 | 38.360 | 39.370 | 38.944 | 13700 | 0.004 | 0.015 |
| **S2** | 52.906 | 45.593 | 59.843 | 45.991 | 8075 | 0.057 | 0.114 |
| **S3** | 22.362 | 45.198 | 19.685 | 45.866 | 12262 | 5.60E-07 | 0.000005 |
| **S4** | 25.054 | 43.680 | 19.685 | 43.356 | 3512 | 8.73E-05 | 0.00044 |
| **S5** | 50.537 | 50.896 | 59.843 | 50.537 | 1266 | 0.598 | 0.906 |
| **S6** | 43.101 | 48.239 | 39.37 | 48.322 | 18447 | 0.033 | 0.0813 |
| **S7** | 39.764 | 44.877 | 39.764 | 43.622 | 2170 | 0.809 | 0.9063 |
| **S8** | 51.575 | 46.453 | 49.606 | 45.082 | 3346 | 0.906 | 0.9063 |
